# Economic value and characteristics of cloud pharmacy for children based on internet hospital in western China during the COVID-19 pandemic: Cross-sectional survey study

**DOI:** 10.3389/fpubh.2022.1034450

**Published:** 2022-11-03

**Authors:** Bin Yang, Qiang Wen, Yi Zhang, Xiuling Wang, Xiangdong Yin, Qianbo Li, Qinling Li, Lin Song

**Affiliations:** ^1^Department of Pharmacy, Children's Hospital of Chongqing Medical University, Chongqing, China; ^2^Ministry of Education Key Laboratory of Child Development and Disorders, Chongqing, China; ^3^National Clinical Research Center for Child Health and Disorders, Chongqing, China; ^4^Chongqing Key Laboratory of Pediatrics, Chongqing, China; ^5^Department of Information, Children's Hospital of Chongqing Medical University, Chongqing, China; ^6^Department of Internet Hospital Office, Children's Hospital of Chongqing Medical University, Chongqing, China

**Keywords:** cloud pharmacy, economic value, online prescription, internet hospital, children, western China, COVID-19

## Abstract

**Background:**

Online health care services have been encouraged by the Chinese government in recent years, and the COVID-19 pandemic catalyzed the rapid growing of internet hospitals. As an integral part of online health care services, little is known about the economic value and characteristics of cloud pharmacy especially for children. This study aimed to reveal the economic value and comprehensive characteristics of pediatric cloud pharmacy during the COVID-19 pandemic in a tertiary children's hospital in western China.

**Methods:**

A total of 33,254 online prescriptions over the course of February 2020 through December 2021 were analyzed with respect to the user profiles, diseases, consulting behaviors, distribution of departments, delivery region and distance, drug information and degree of satisfaction. The cost savings for patients calculated lost wages and the high-speed railway fees for transport to and from hospital.

**Results:**

A total of 33,254 prescriptions, including 56,216 drugs were delivered to 27 provinces and municipalities of China. The internet cloud pharmacy saved a total of more than RMB 11.17 million in financial costs for patients. Of the 33,254 delivered prescriptions, 50.40% were sent to Chongqing Province, the top 5 provinces for out-of-province prescription deliveries were Sichuan (37.77%), Guizhou (8.00%), Yunnan (1.18%), Hubei (0.66%) and Guangdong (0.42%). In terms of department distribution, neurology (31.7%), respiratory (15.0%) and endocrinology (14.6%) were the top three departments. Epilepsy (16.2%), precocious puberty (10.3%) and asthma (8.7%) were the top three frequently consulted diseases. The peak times of day for online prescriptions occurred at 9 AM and 8 PM. 99.67% of users gave full marks for their internet counseling.

**Conclusion:**

The pediatric cloud pharmacy is efficient, cost-saving and convenient for children with chronic disease or mild symptoms during the COVID-19 pandemic. The widespread use of this pediatric cloud pharmacy can help alleviating pressure on offline hospitals and facilitated people's lives beyond geographical and time-related limitations. Further efforts are needed to be made to improve the quality and acceptance of pediatric cloud pharmacy, as well as to regulate and standardize the management of this novel online health care service.

## Introduction

The coronavirus disease 2019 (COVID-19) rampantly swept across the entire globe in 2020 (1). The Chinese government has implemented a number of preventive and quarantine policies to limit the spread of the epidemic, including diagnosis at an early stage, symptomatic monitoring of contacts with suspected and confirmed cases, restrictions on people's movement, reduced transportation, social distancing and community-based isolation (2). During the COVID-19 pandemic, the strict quarantine policy deterred most public access to offline health care (3). For patients with chronic diseases requiring the long-term use of medications, at-home quarantine without consecutive medication could lead to disease exacerbation. Therefore, the management of patients with chronic diseases has become a crucial issue with the large-scale outbreaks of COVID-19 (4, 5).

China has the largest population, but medical care resources are relatively limited and geographical distribution are imbalanced. High quality medical resources and services are concentrated in large cities, and most of the top 100 comprehensive hospitals are located in economically well-developed regions (6). The pediatric medical resources are more scarce, there are only 4 pediatricians per 10,000 children in China (7). To counter the problems mentioned above, the Chinese government has enacted various relevant policy interventions to develop the internet plus medical and health care plan by promoting the integration of the internet and medical care to increase access to health care and improve the quality and efficiency of health care (8). Internet hospitals are an important part of internet plus medical and health care plan. The COVID-19 pandemic has spawned the explosive growth of internet hospitals (9). Internet hospitals, in general, provide a variety of online services directly to patients including booking appointments, checking test results, online prescriptions, health education, and follow-up consultations (9). The ever-increasing popularity in adoption of smartphones and tablet computers with an internet penetration rate of 70.4% makes internet medical care model which allows patients to access information, assessments, and treatments in a timely manner, was accessible to the public (10, 11).

On January 21, 2020, Children's Hospital of Chongqing Medical University has officially opened the internet hospital by WeChat public platform. In the beginning, the internet hospital provided only web-based services for returning patients and online consultation service for COVID-19. In order to provide better and convenient online medical services for pediatric patients, on February 13, 2020, our hospital set up the cloud pharmacy based on the internet hospital. This cloud pharmacy would enable remote services including web-based consultation, prescription, and home delivery of medications.

The key values of online medical services are saving patients' and their families' time (8). But no one has studied how much economic value the internet hospital online medical services have saved patients specifically. The novelty of the current study might be the long 2-year period which could further identify pediatric patients' behavior patterns and economic value of the cloud pharmacy. To explore the advantages of cloud pharmacy for pediatric patients during the COVID-19 pandemics, we analyzed the prescriptions of online outpatients at the Children's Hospital of Chongqing Medical University in Chongqing city, in underdeveloped western China. Data from February 2020 through December 2021 were collected to reveal the economic value, characteristics, acceptance, and initial impact of the cloud pharmacy.

## Methods

### Data collection

The detailed information of online prescriptions was obtained automatically from the hospital information system (HIS) of the Children's Hospital of Chongqing Medical University from February 2020 through December 2021. The collected data included patients' gender, age, diagnose, department, drug information including generic name and quantity, time initiation for the online prescriptions, dialogue rounds between physician and patient, drug delivery region, and the satisfaction score (1–5, 5 being the best).

### Data cleaning

The total number of online prescriptions from the HIS was 38,519 between February 2020 and December 2021. We excluded 5,265 individuals with missing information before the analyses. Four thousand eight hundred sixty-seven were missing mailing address, 322 were missing prescribed medication, and 76 were missing patient gender. After the data-filtering steps, there were 33,254 online prescriptions remaining.

### Data processing

The disease diagnosis was encoded according to the International Classification of Diseases, version 10 (ICD-10). Generally, the diagnosis was standardized as one-by-one mapping to ICD-10. For prescription consisted of multiple diagnoses, we split it into multiple samples to calculate the diagnosis distribution, and each sample with a distinct diagnosis. Drug delivery distance was calculated from the hospital to the mailing destination.

### Economic value calculation

We calculated the cost savings for patients in terms of time cost and costs for transport to and from hospital. In this study, we converted the time cost into lost wages for the parents of the children. The cost for transport to and from hospital is based on the fare of the second-class of the high-speed railway. The round-trip transportation cost is calculated bilaterally for the parents and the child (half ticket). The formula for the economic cost savings per patient was as follows: wage loss+1.5^*^2^*^ train ticket fee.

### Statistical analysis

We used Microsoft Excel 2019 (Microsoft Corp., Redmond, WA, USA) and SPSS 19.0 (SPSS Inc, IL, USA) for data storage and analysis. Data were collected as continuous and categorical variables. Continuous variables are described using the means ± standard. For categorical data, frequencies and percentages were used.

### Ethics statement

This retrospective study was approved by the Ethics Committee of the Children's Hospital of Chongqing Medical University (Ethics Approval Number 2022-348).

## Results

### Flowchart of online prescriptions services

**Figure 1:**
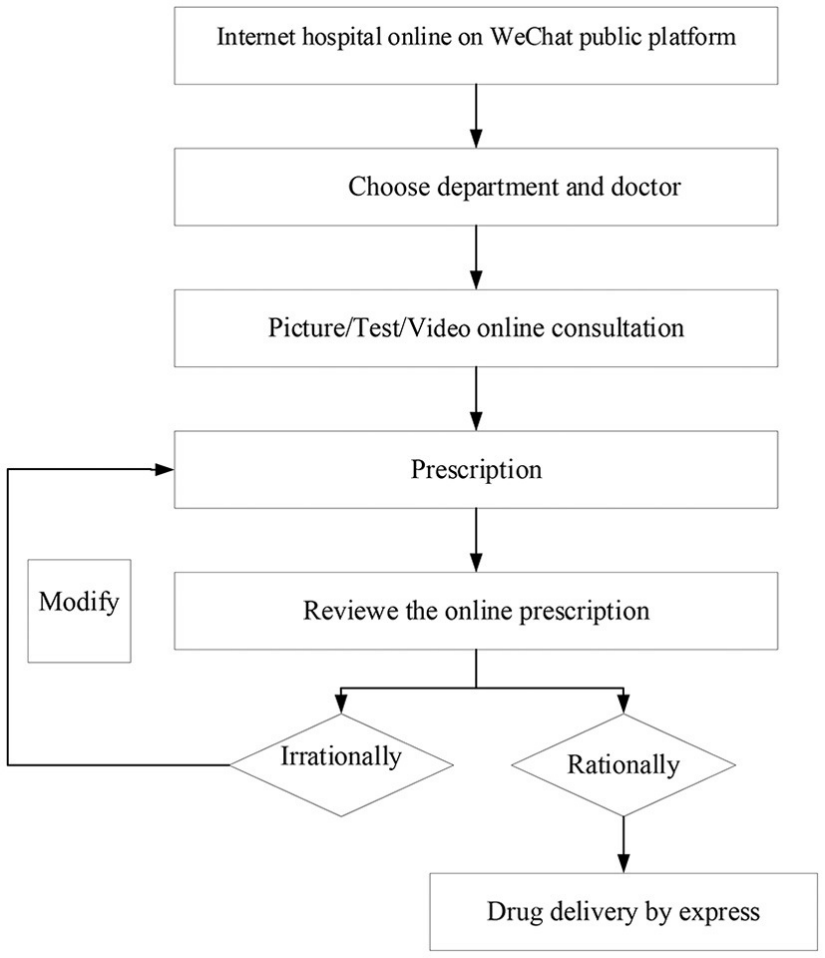
Flowchart of online prescriptions services.

The cloud pharmacy services are provided 24 h per day. Physicians used their time outside of routine working hours to provide online services. Figure 1 shows the flowchart of online prescriptions services. The patient who had a medical record at our hospital during the past 3 months chose a department and physician using the WeChat public platform *via* a mobile phone, then filled in the medical record. Patients waited for the physician's picture/test/video consultation. As the diagnosis was completed, the physician made an online prescription. Then the pharmacist reviewed the online prescription with electronic signature (CA). If the prescribing was irrationally, the clinician must modify the prescription. After fee was paid, the drugs were delivered to the patient's home by express delivery.

### Patient demographics and characteristics of online prescriptions

The patient demographics are shown in Table 1. During the study period, there were 33,254 patients who received prescription in total. 54.60% (18,157/33,254)patients were male. The mean age of the patients was 6.65 years. The largest group of patients were school age (*n* = 16,212, 48.75%), followed by those who were preschool age (*n* = 9,550, 28.72%).

**Table 1:** Baseline characteristics of patients (*N* = 33,254).

**Characteristic**	**Value**
**Sex**, ***n*** **(%)**	
Male	18,157 (54.60%)
Female	15,097 (45.40%)
**Age (years)**	
Median (SD)	6.65 (3.41)
**Group**, ***n*** **(%)**	
Neonatal and infancy	1,229 (3.70)
Toddler's age	3,856 (11.60)
Preschool age	9,550 (28.72)
School age	16,212 (48.75)
Adolescence	2,407 (7.24)

**Figure 2:**
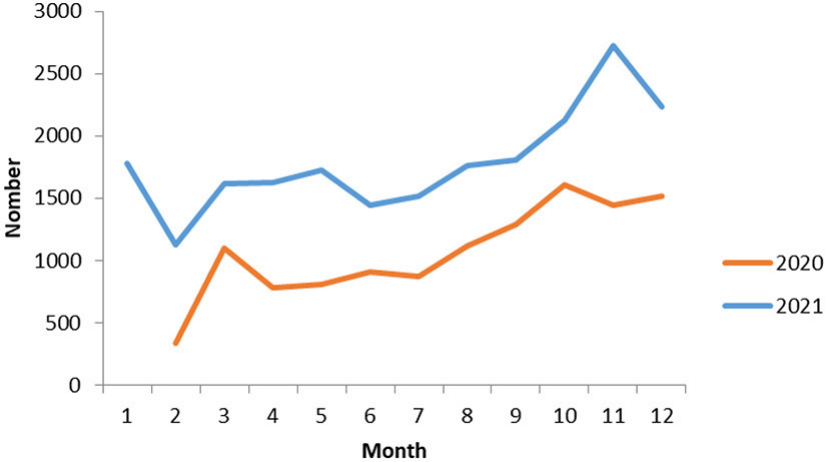
Monthly number of the online prescriptions.

A total of 33,254 online prescriptions were delivered between February 2020 and December 2021. The monthly number of the online prescriptions was 1,446 as showed in Figure 2. From 2020 to 2021, the number of online prescriptions was increased by 82.46%.There was an increase in the use of the online pharmacy service with time.

**Figure 3:**
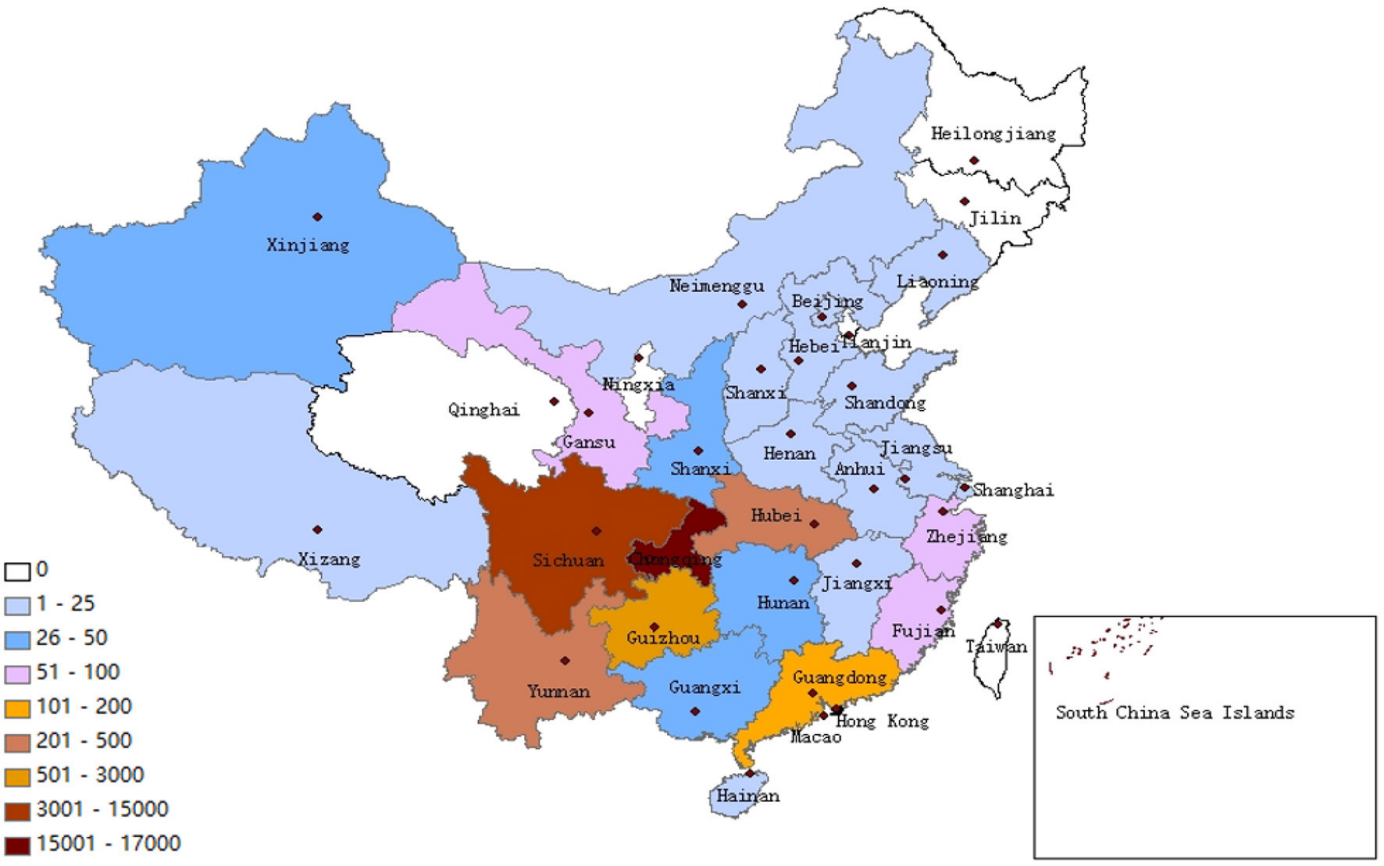
Regional distribution of prescription deliveries.

The 33,254 delivered prescriptions were delivered to 27 provinces and municipalities of China. 50.40% (16,759/33,254) of the online prescriptions were sent to Chongqing Province, the top 5 provinces for out-of-province prescription deliveries were Sichuan (*n* = 12,561, 37.77%), Guizhou (*n* = 2,659, 8.00%), Yunnan (*n* = 391, 1.18%), Hubei (*n* = 219, 0.66%), Guangdong (*n* = 139, 0.42%) (Figure 3). Internet prescriptions in Chongqing and the neighboring provinces (Sichuan, Guizhou, Hubei, Shaanxi, Hunan) accounted for 97.11% (32,292/33,254)of total prescriptions.

**Figure 4:**
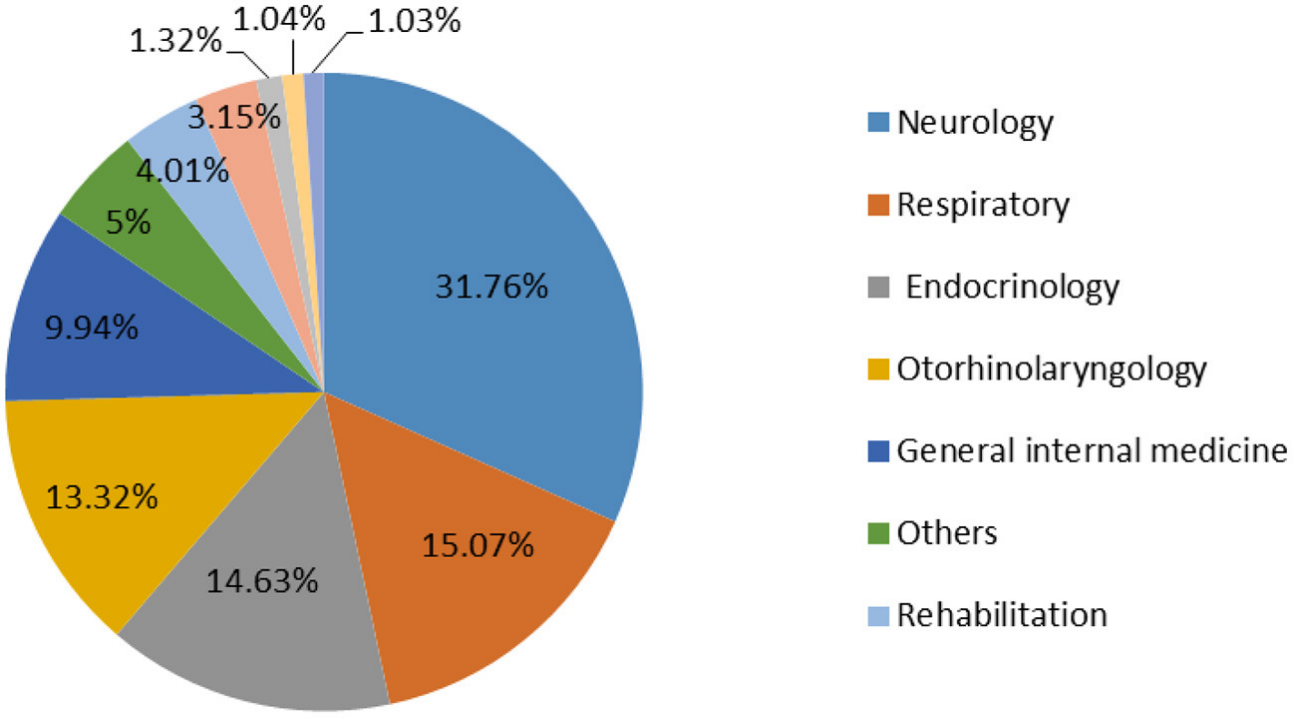
Distribution of the top 10 departments for the online prescriptions.

There were 28 departments on the internet hospital, Figure 4 showed the most popular online prescription departments were neurology (*n* = 10,561, 31.76%), respiratory (*n* = 5,012, 15.07%) and endocrinology (*n* = 4,865,14.63%). 45,380 diagnoses were represented in our data set. The top 10 diseases are illustrated in Table 2 and they accounted for 63.94% (29,018/45,380) of the total diagnoses.

**Table 2:** The top 10 diseases for the online prescriptions.

**Diagnosis**	**Value (*n* = 45,380) (*n*%)**
Epilepsy	7,755 (17.09)
Asthma	4,686 (10.33)
Precocious puberty	4,508 (9.93)
Tic disorder	2,793 (6.15)
Allergic rhinitis	2,620 (5.77)
Attention deficit	1,656 (3.65)
Recurrent respiratory infection	1,456 (3.21)
Adenoid hypertrophy	1,423 (3.14)
Rhinitis	1,295 (2.85)
Intellectual disability	826 (1.82)

**Figure 5:**
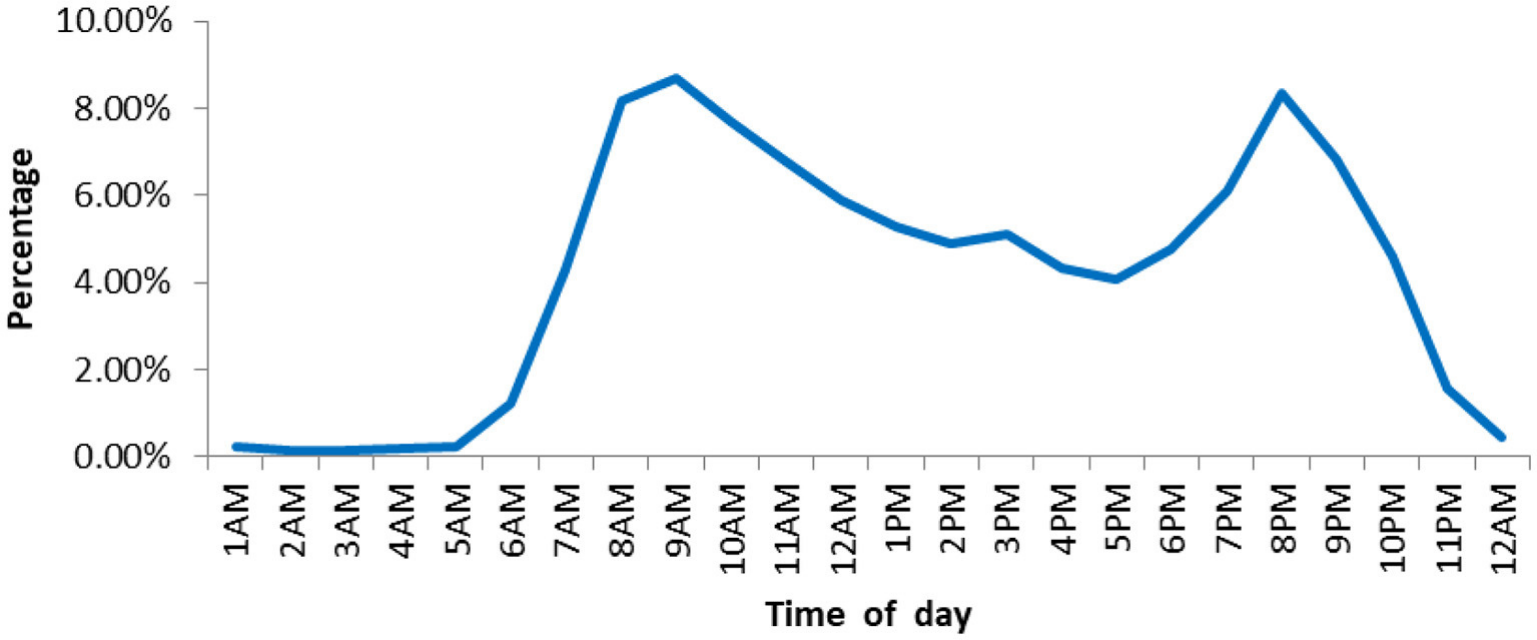
Distribution of times of day for online prescription.

Figure 5 showed the times of day when users began to seek for online prescriptions, there were two peaks regarding times of day for online prescription, and the two peak times were 9AM (8.68%, 2,888/33,254) and 8 PM (8.35%, 2,776/33,254).

**Figure 6:**
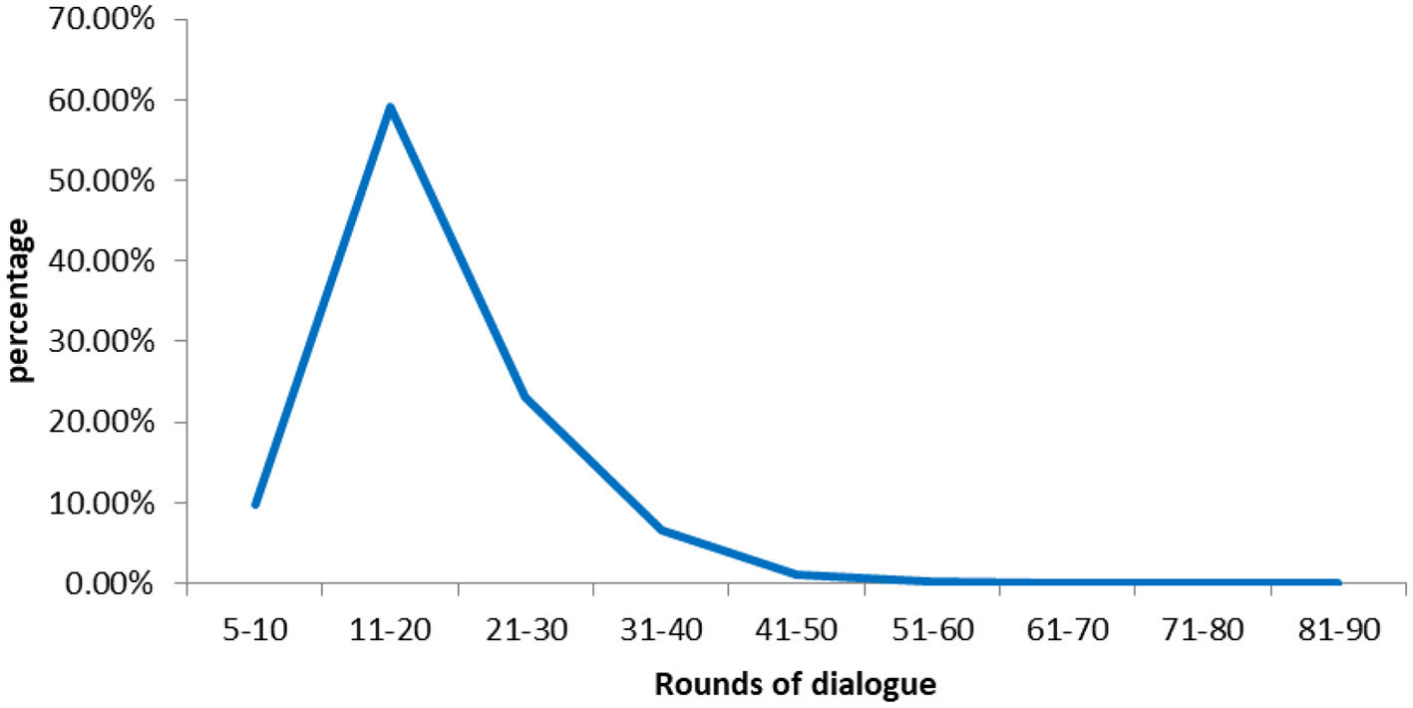
Distribution of rounds of dialogue between physicians and users.

Figure 6 showed the distribution of rounds of dialogue between the physicians and patient parents. The number of rounds of dialogue peaked at around the 11–20 range. The average number of the rounds of dialogue between physicians and patient parents were 18.34.

For the 33,254 delivered prescriptions, the number of medicines were 56,216,the top 10 delivered are listed in Table 3. The top 10 medicines accounted for 34.99% (19,672/56,216) of the total medicines. The average amount of drugs per online prescriptions was RMB 277.09 and the range of drugs per prescription varied from RMB 0.09 to RMB 4,457.09. Most of the medicines were used to treat endocrinopathies, neurologic, respiratory and otorhinolaryngology diseases, which was consistent with the department distribution of online prescription.

**Table 3:** Top 10 delivered medicines.

**Drug scientific name**	**No (%)**	**Pharmaceutical dosage form**
Dabuyin pill	4,290 (7.63)	Bolus
Montelukast	3,355 (5.97)	Chewable tablet
Sodium valproate	2,063 (3.67)	Solution
Fluticasone propionate	1,991 (3.54)	Aerosol
Pediatric intelligence syrup	1,973 (3.51)	Solution
Levetiracetam	1,942 (3.45)	Tablet
Mometasone furoate aqueous nasal spray	1,448 (2.04)	Spray
Tiapride hydrochloride	1,448 (2.04)	Tablet
Lysine, inosite and vitamin B_12_ oral solution	1,162 (2.07)	Solution
Oxcarbazepine	1,076 (1.91)	Tablet

## Economic value analysis of cloud pharmacy

The average mailing distance was 200.96 km. The average annual wage of the employed persons in private urban units nationwide in 2020 and 2021 is RMB 57,727 and RMB 62,884 in China (12, 13), and the daily wage is RMB 158 and RMB 172 in 2020 and 2021, respectively. The ticket price of high-speed rail for second-class seat is RMB 0.30855 per kilometer (14). For patients with a median distance of 50 km from home to hospital, a round trip to hospital usually takes 0.5 day,100–500 km takes one day,500–750 km takes 1.5 days, 750 km to 1,500 km takes 2 days and 1,500–3,000 km takes 3 days. For example, in 2021, a patient living in Chengdu, which is 300 km from our hospital, the wage loss is calculated as RMB 172 for 1 day, the round-trip transportation cost is RMB 277.70 (1.5^*^2^*^0.30855^*^300 = 277.70), the direct economic cost savings of the cloud pharmacy for this patient is RMB 449.70 (172 + 277.70 = 449.70). In 2 years, internet cloud pharmacy has saved patients a total of more than RMB 11.17 million in financial costs.

### Users' satisfaction with online prescriptions

Among the 33,254 online prescriptions, there were 33,125 that had satisfaction scores. A total of 99.67% (33,016/33,125) of these satisfaction scores were 5, suggesting that most majority of the users had a good experience of the online prescriptions.

## Discussion

### Principal findings

The infection spread of COVID-19 is mainly through respiratory droplets and direct contact with infected individuals (15). Hospitals are not only important battlefields for pandemic prevention and treatment, they are also high-risk places for epidemic transmission. Traditional hospitals require patients to visit the hospital to obtain medications, which lead to potential infection and deterioration of the patient's health, particularly for these with chronic disease during the epidemic. On the other hand, patients in rural or remote areas could have problems finding the exact prescription medications they need especially for these with chronic diseases, as those drugs might be not available in local hospitals. The cloud pharmacy based on telemedicine launched by Children's Hospital of Chongqing Medical University made it easy for patients cross the country to access online prescription medications in a secure, rapid, cost-saving and convenient way without cross-contamination caused by close contact in hospitals during COVID-19 pandemic.

Some scholars have researched the characteristics of internet cloud pharmacy. Liang Ding et al. and Zhuo-Jia Chen et al. investigated the characteristics, acceptance, and initial impact of cloud pharmacy during the outbreak of COVID-19 in the economically developed south China (16, 17). Hammour and colleagues in Jordan studied how well the internet pharmacy worked during the COVID-19 pandemic (18). These researches were all performed in adults hospitals and were not targeted for children. Compared to the above studies, the investigation we included was up to 2 years and we had a whopping 33,254 pieces of research data. The long 2-year period and extensive research data could get an in-depth analysis of pediatric patients' behavior patterns on cloud pharmacy.

In previous studies, the most important feature of internet health care, i.e., cost saving for patients, remained at the theoretical level, without actual data to support it (8). As far as we know, this is the first study to calculate how much economic value the cloud pharmacy has saved for patients. There is more than 35,000 km of high-speed railway passenger lines in China, accounting for about 70% of all high-speed railways in operation worldwide (19). In this study we could calculate the round-trip travel costs as the high speed train ticket. The costs of lost wages and the round-trip travel costs have saved the patients more than RMB 11.17 million during the 2 years. These results will serve as a useful reference for policymakers to support the development of internet healthcare in the COVID-19 pandemic.

### Policy recommendations

The COVID-19 has become the most dangerous threat to health, economic of rural poverty (20) and food security (21) in recent history. As of 5 October, 2022, 616,427,419 individuals in the world have been infected, of whom 6,528,557 have died (22). Most countries have developed policies to control the disease (23). Healthcare systems use telemedicine as the most effective tool to combat COVID-19 outbreaks (24). For example, China's government has encouraged internet hospitals to join the prevention and control of the COVID-19 outbreak (25). In the USA, emergency supplemental funding legislation was passed to support COVID-19 research and expansion of telemedicine in metropolitan areas (26). In pandemics, telemedicine can provide services for prevention, screening, diagnosis, triage, treatment and follow-up (27). Based on our findings, there are some suggestions that deserve further discussion to provide policy enlightenment for the development of cloud pharmacy.

First, the main work of the internet cloud pharmacy is not only to deliver drugs to patients. Pharmacists should use the platform to provide patients with pharmaceutical care such as online medication risk assessment, optimal medication design, adverse drug reaction management and chronic disease management.

Second, internet drug delivery modes should be diverse, in addition to mailing drugs from hospitals, not only can patients pick up their medicines from hospitals or pharmacies, but the medicines also can be delivered from the manufacturer's stock house.

Third, in the process of online health care services, it's required to exchange data between hospitals, patients and the third-party companies which will result in privacy leakage. But this facet of regulation is still blank. It is important to introduce laws and regulations as soon as possible to protect personal privacy and data security for the online health care services.

Fourth, it is important that the development of cloud pharmacy should be built on the premise that telemedicine becomes a mainstream component of our health system.

### Limitations

In terms of economic value, this study only calculated the lost wages for the parents of the children and round-trip travel costs. The data for costs of accommodation, food, and nucleic acid monitoring for cross-province patients were not available. Not including these data would underestimate the economic value of internet hospitals. But for countries where high-speed railway transportation is not developed, this method of using high-speed rail fares as round-trip transportation tolls is not desirable. This will limit the worldwide application of this method for calculating cloud pharmacy's economic value.

### Future prospects

Partially and irreversibly, the cloud pharmacy is integrating into traditional medicine. People's lifestyle habits might change as a result of this once-in-a-century pandemic of COVID-19, particularly in terms of health management. Therefore, more effort is needed to make better use of the cloud pharmacy service for children based on internet hospital during and after the current pandemic. In the future, under the national health department support, a regional internet prescription flow platform based on our cloud pharmacy targeted for children can be constructed to achieve the goal that sharing internet prescription information.

## Conclusion

To the best of our knowledge, this was the first report to evaluate the economic value and characteristics of cloud pharmacy for children based on internet hospital in western China from December 2020 to December 2021 during the COVID-19 pandemic. The cloud pharmacy can facilitate people's lives beyond geographical and time-related limitations in an efficient, cost-saving and convenient way for children with chronic disease or mild symptoms during the COVID-19 pandemic. The data about how much money has been saved for patients in this study will serve as a useful reference for policymakers to support the development of internet healthcare in the COVID-19 pandemic.

Currently, the medical supervision for service quality, content, fees, compensation mechanisms and service provider qualifications for online health care service is still in its infancy and remains exploratory. And further efforts are needed to be made to improve the quality and acceptance of pediatric cloud pharmacy, as well as to regulate and standardize the management of this novel online health care service.

## Data availability statement

The raw data supporting the conclusions of this article will be made available by the authors, without undue reservation.

## Ethics statement

This retrospective study was approved by the Ethics Committee of the Children's Hospital of Chongqing Medical University.

## Author contributions

QinL and LS: conceptualization. QW and XW: data curation. BY: writing—original draft. QW: writing—review and editing. YZ: formal analysis. XY and QiaL: methodology. All authors have read and agreed to the published version of the manuscript.

## Conflict of interest

The authors declare that the research was conducted in the absence of any commercial or financial relationships that could be construed as a potential conflict of interest.

## Publisher's note

All claims expressed in this article are solely those of the authors and do not necessarily represent those of their affiliated organizations, or those of the publisher, the editors and the reviewers. Any product that may be evaluated in this article, or claim that may be made by its manufacturer, is not guaranteed or endorsed by the publisher.
